# Paragraphs

**Published:** 1882-05

**Authors:** 


**A Sweet Philosophy.**—The celebrated teacher, Rabbi Meir, sat during the whole of one Sabbath-day in the public school, instructing the people. During his absence from the house, his two sons died—both of them of uncommon beauty, and enlightened in the law. His wife bore them to her bed chamber, laid them upon the marriage-bed, and spread a white covering over their bodies. In the evening the Rabbi came home.

She reached him a goblet. He praised the Lord at the going out of the Sabbath, drank, and asked:

"Where are my two sons, that I may give them my blessing? I repeatedly looked round the school, and I did not see them there. Where are my sons, that they, too, may drink of the cup of blessing?"

"They will not be far off," she said, and placed food before him, that he might eat.

He was in a gladsome and genial mood; and when he had said grace after the meal, she thus addressed him:

"Rabbi, with thy permission I would fain propose to thee one question."

"Ask it, then, my love," he replied.

"A few days ago a person intrusted some jewels to my custody; now he demands them again. Should I give them up?"

"This is a question," said Rabbi Meir, "which my wife should not have thought it necessary to ask. What! wouldst thou hesitate or be reluctant to restore to every one his own?"

"No," she replied; "but yet I thought it best not to restore them without acquainting thee therewith."

She then led him to the chamber, and stepping to the bed, took the white covering from the dead bodies.

"Ah! my sons, my sons!" loudly lamented the father. "My sons, the light of my eyes, and the light of my understanding! I was your father, but you were my teachers in the law."

The mother turned away and wept bitterly. At length she took her husband by the hand and said: "Rabbi, didst thou not teach me that we must not be reluctant to restore that which was intrusted to our keeping? See, the Lord gave, and the Lord has taken away, and blessed be the name of the Lord."

"Blessed be the name of the Lord!" echoed Rabbi Meir; "and blessed be his name for thy sake, too; for well it is written: 'Whoso hath found a virtuous wife hath a greater treasure than costly pearls. She openeth her mouth with wisdom, and in her tongue is the law of kindness.'"

**How to Admonish.**—We must consult the gentlest manner and softest seasons; for advice must not fall like a violent storm, bearing down and making those to droop whom it is meant to cherish and refresh. It must descend as dew upon the tender herb, or like melting flakes of snow; the softer it falls, the longer it dwells upon and deeper it sinks into the mind. If there are few who have the humility to receive advice as they ought, it is often because there are as few who have the discretion to convey it in a proper vehicle, and to qualify the harshness and bitterness of reproof, against which corrupt nature is apt to revolt, by an artful mixture of sweet and pleasant ingredients. To probe the wound to the bottom, with all the boldness and resolution of a good spiritual surgeon, and yet with all the delicacy and tenderness of a friend, requires a very dexterous and masterly hand. An affable deportment and a complacency of behavior will disarm the most obstinate. Whereas, if, instead of pointing out their mistake, we break out into unseemly sallies of passion, we cease to have any influence over them, or rather create a feeling antagonistic to the advice we wish to give them.

**Prejudice.**—All men are apt to have a high conceit of their own understanding, and to be tenacious of the opinions they profess; and yet almost all men are guided by the understandings of others, not by their own, and may be said more truly to adopt than to beget their opinions. Nurses, parents, pedagogues, and after them all, and above them all, that universal pedagogue system fills the mind with notions which it has no share in framing; which it receives as passively as it receives the impressions of outward objects, and which, left to itself, it would never have framed, or would have examined afterwards. Thus prejudices are established by education, and habits by custom. We are taught to think what others think, not how to think for ourselves; and whilst the memory is loaded, the understanding remains unexercised, or exercised in such trammels as constrain its motions and direct its pace, till that which is artificial becomes in some sort natural, and the mind can go to no other. It may sound oddly, but it is true in many cases, to say, that if men had learned less, their way to knowledge would be shorter and easier. It is, indeed, shorter and easier to proceed from ignorance to knowledge than from error. They who are in the last must unlearn before they can learn to any good purpose; and the first part of this double task is not in many respects the least difficult, for which reason it is seldom undertaken.

**How Ladies should Dress.**—As you look from your windows in Paris, observe the first fifty women who pass; forty have noses depressed in the middle, a small quantity of dark hair, and a swarthy complexion. But, then, what a toilet! Not only suitable for the season, but the age and complexion of the wearer. How neat the feet and hands! How well the clothes are put on, and more than all, how well they suit each other!

Before English women can dress perfectly, they must have the taste of the French, especially in color. One reason why we see colors ill-arranged in England is that the different articles are purchased each for its own imagined virtues, and without any thought of what is to be worn with it. Women, while shopping, buy what pleases the eye on the counter, forgetting what they have at home. That parasol is pretty, but it will kill, by its color, one dress in the buyer's wardrobe, and be unsuitable for the others. To be magnificently dressed costs money; but to be dressed with taste is not expensive. It requires good taste, knowledge, and refinement. Never buy an article unless it is suitable to your age, habit, style, and the rest of your wardrobe. Nothing is more vulgar than to wear costly laces with a common delaine, or cheap lace with expensive brocades.

What colors, it may be asked, go best together? Green with violet; cold with dark crimson, or lilac; pale blue with scarlet; pink with black or white; and gray with scarlet or pink. A cold color generally requires a warm tint to give life to it. Gray and pale blue, for instance, combine well, both being cold colors. White and black are safe wear, but the latter is not favorable to dark or pale complexions. Pink is to some skins the most becoming; not, however, if there is much color in the cheeks and lips, and if there be even a suspicion of red in either hair or complexion. Peach color is, perhaps, one of the most elegant colors worn. Maize is very becoming, particularly to persons with dark hair and eyes. But whatever the colors or materials of the entire dress, the details are all in all: the lace around the bosom and sleeves, the flowers—in fact, all that furnishes the dress. The ornaments in the head must harmonize with the dress. If trimmed with black lace, some of the same should be worn in the head, and the flowers, which are worn in the hair, should decorate the dress.

**Ingratitude to Parents.**—There was once a father who gave up every thing to his children—his house, his fields, and goods—and expected that for this his children would support him. But after he had been some time with his son, the latter grew tired of him, and said to him: "Father, I have had a son born to me this night, and there, where your arm-chair stands, the cradle must come. Will you not, perhaps, go to my brother, who has a larger room?'

After he had been some time with the second son, he also grew tired of him, and said: "Father, you like a warm room, and that hurts my head. Won't you go to my brother, the baker?" The father went, and after he had been some time with the third son, he also found him troublesome, and said to him: "Father, the people run in and out here all day, as if it were a pigeon-house, and you can not have your noonday sleep. Would you not be better off at my sister Kate's, near the town-wall?"

The old man remarked how the wind blew, and said to himself: "Yes, I will do so; I will go and try it with my daughter. Women have softer hearts." But after he had spent some time with his daughter, she grew weary of him, and said she was always so fearful when her father went to church, or any where else, and was obliged to descend the steep stairs, and at her sister Elizabeth's there were no stairs to descend, as she lived on the ground-floor.

For the sake of peace the old man assented, and went to his other daughter. But after some time she, too, was tired of him, and told him, by a third person, that her house near the water was too damp for a man who suffered with gout, and her sister, the grave-digger's wife, at St. John's, had much drier lodgings. The old man himself thought she was right, and went outside the gate to his youngest daughter, Helen. But after he had been three days with her, her little son said to his grandfather: "Mother said yesterday to cousin Elizabeth, that there was no better chamber for you than such a one as father digs." These words broke the old man's heart, so he sank back in his chair and died.—*Martin Luther.*

**Entering a Room.**—I have sometimes envied the coolness and self-possession of those gentlemen who, fortified by long practice, can enter a drawing-room, having no previous knowledge of its inmates, with as much *sangfroid* and indifference as if they were lounging into a box at the opera, and commence a conversation without exhibiting the slightest embarrassment. Yet, after all, I doubt whether they are to be envied, for I apprehend that such demeanor must be the result either of remarkable self-complacency or of callousness of the heart and imagination. It argues the absence, I think, of that chivalrous feeling toward the fair sex which in the middle ages was carried to so extreme a length that, in the words of an old writer of romance, "a true knight should stand more awed and abated in the presence of beauty than if he were summoned before the throne of the most puissant emperor of the world."—*Norman Sinclair.*

**A Good Woman Never Grows Old.**—Years may pass over her head, but if benevolence and virtue dwell in her heart, she is cheerful as when the spring of life opened to her view. When we look at a good woman we never think of her age; she looks charming as when the rose of youth first bloomed on her cheek. That rose has not faded yet; it will never fade. In her neighborhood she is the friend and benefactor. Who does not respect and love the woman who has passed her days in acts of kindness and mercy? We repeat, such a woman can never grow old. She will always be fresh and buoyant in spirits, and active in humble deeds of mercy and benevolence.

**Flowers for Winter.**—Flowers intended for winter blooming need a season of repose, especially tropical plants, such as geranium, fuchsia, etc., which should be allowed rest from growth during the months of July and August, by almost entirely withdrawing the supply of water. Of course the leaves will fall off, but the plants will be fitted to start into fresh and vigorous growth as soon as the water is again supplied. Previous to this, the branches of the fuchsia should be pruned in, and water given sparingly at first, increasing the supply as the young shoots grow.

### NEVER

Never taste an atom when you are not hungry; it is suicidal.

Never enter an omnibus without having the exact change.

Never stop to talk in a church aisle after service is over.

Never hire servants who go in pairs, as sisters, cousins, or any thing else.

Never blow your nose between your thumb and fingers.

Never deposit the results of a "hawk" or cough on the sidewalk.

Never pick your nose and look at it.

Never open your handkerchief to inspect the product of a "blow."

Never speak of your father as "the old man."

Never reply to the epithet of a drunkard, a fool, or a fellow.

Never speak contemptuously of womankind.

Never abuse one who was once your bosom-friend, however bitter now.

Never smile at the expense of your religion or your Bible

Never stand at the corner of a street.

Never take a second nap.

Never eat a hearty supper.

Never insult poverty.

Never eat between meals.

